# Multiparametric whole-body magnetic resonance imaging in breast cancer management: A prospective study

**DOI:** 10.1177/03008916261447104

**Published:** 2026-06-04

**Authors:** Michela Palleschi, Filippo Merloni, Andrea Prochowski Iamurri, Emanuela Scarpi, Danila Diano, Caterina Gianni, Domenico Barone, Giandomenico Di Menna, Chiara Casadei, Roberta Maltoni, Ugo De Giorgi, Antonino Musolino, Alice Rossi

**Affiliations:** 1 Medical Oncology, Breast & GYN Unit, IRCCS Istituto Romagnolo per lo Studio dei Tumori (IRST) “Dino Amadori”, Meldola, Italy; 2 Radiology Unit, IRCCS Istituto Romagnolo per lo Studio dei Tumori (IRST) "Dino Amadori", Meldola, Italy; 3 Biostatistics and Clinical Trials Unit, IRCCS Istituto Romagnolo per lo Studio dei Tumori (IRST) "Dino Amadori", Meldola, Italy; 4 Outcome Research, Healthcare Administration, IRCCS Istituto Romagnolo per lo Studio dei Tumori (IRST) "Dino Amadori", Meldola, Italy; 5 Department of Medical Oncology, IRCCS Istituto Romagnolo per lo Studio dei Tumori (IRST) "Dino Amadori," Meldola, Italy; 6 Department of Medical and Surgical Sciences, University of Bologna, Bologna, Italy

**Keywords:** whole-body magnetic resonance, breast cancer, oligometastastic breast cancer, bone metastases, lobular breast cancer

## Abstract

Multiparametric Whole-Body Magnetic Resonance Imaging (WB-MRI) may enhance bone metastasis detection in metastatic breast cancer (mBC) patients. We report preliminary results from a prospective clinical trial investigating WB-MRI utility in BC patients with bone-predominant lesions, equivocal imaging findings or oligometastatic disease (OMD). Fifty-nine consecutive patients underwent baseline staging with standard imaging techniques (SIT), followed by WB-MRI. WB-MRI led to treatment-plan modifications in 40% of cases; 83% of lobular carcinoma cases exhibited additional metastases on WB-MRI. Among the 23 patients who underwent at least one reassessment by WB-MRI and SIT, the concordance rate was 69.6%. Among 18 OMD patients, WB-MRI led to the redefinition of the disease status in 44.4% of cases. These findings underscore the value of WB-MRI in BC.

## Introduction

Metastatic breast cancer (mBC) is a complex and heterogeneous malignancy, requiring comprehensive imaging techniques to stage and monitor the progression of the disease accurately.^1^ Standard imaging techniques (SIT), such as computed tomography (CT), fluorine-18 fluorodeoxyglucose (FDG) positron emission tomography with computed tomography (PET/CT), and bone scintigraphy (BS), have limitations in detecting bone metastases. For example, the development of new osteosclerotic lesions should not be classified as progression unless there is evidence of disease progression and there are no objective CT density measurements for disease assessment.^2-6^

The use of multiparametric Whole-Body Magnetic Resonance Imaging (WB-MRI) may improve the accuracy of detecting bone metastases and potentially impact the management of breast cancer (BC), especially in lobular neoplasms.^7^ WB-MRI has shown higher accuracy in detecting bone metastases when used alongside SIT, also in BC patients. In addition to the advantage of a non-ionizing radiation technique, WB-MRI does not require intravenous contrast agents, making it a safer option, especially in pregnant women.^8^ This imaging method can be especially useful in BC patients with oligometastatic disease (OMD), currently defined by the presence of one to five metastatic lesions that can be safely treated with metastases-directed therapy (MTD).^9^ Data regarding the impact of MDT on survival are conflicting, and the correct management of OMD remains a matter of debate.^10^ Possible explanations include the lack of biological criteria and advanced imaging methods to better define OMD.

Our study investigated the added value of WB-MRI to CT and/or PET/CT for detecting additional metastases or disease progression, and whether this may lead to treatment plan modifications.

## Methods

We report the results of a monocentric prospective clinical trial which is currently ongoing since 2023. WB-MRI was used as a baseline staging tool alongside routine SIT for specific groups of BC patients. Although there is no published specific protocol for WB-MRI in mBC, all WB-MRI were performed on a 3T MRI scanner (Ingenia, Philips, Eindhoven, Netherlands) using a previously published protocol to standardize multiparametric WB-MRI studies.^11-14^

We included patients with bone-predominant lesions, indeterminate imaging findings, or OMD on SIT, or a rise in tumor markers with negative SIT findings. We analyzed discrepancies between WB-MRI and SIT and assessed the impact of WB-MRI on treatment-plan modification and oligometastatic state definition.

Treatment-plan modification was defined as any change in clinical management, including changes in the indication for systemic oncologic therapy, radiotherapy, or invasive diagnostic procedures (e.g., biopsy)

The radiologists who interpreted the WB-MRI were not blinded to the previous SIT. WB-MRI examinations were reviewed by at least two radiologists with more than five years of experience in WB-MRI interpretation.

All discrepancies between SIT and WB-MRI were discussed by a multidisciplinary team of oncologists and radiologists, with particular emphasis on evaluating the additional metastases identified by WB-MRI.

## Results

Fifty-nine consecutive BC patients were assessed with CT of the thorax, abdomen, and pelvis and/or PET/CT followed by WB-MRI. The interval between SIT and WB-MRI was always equal to or less than 21 days. Ductal histology was reported in 27 cases (51.9%) and lobular histology in 18 (34.6%). Other patients have mixed histologies (3.9%), other histologies (9.6%), or unknown histologies (11.9%). Of the patients with suspicious lesions on SIT, 25/59 (42.4%) were reclassified as metastatic upon WB-MRI; in 21 patients (35.6%), WB-MRI led to a modification of the therapeutic approach. The therapeutic decision changed in 11 patients (18.6%) due to the identification of new lesions or progression of known metastatic sites on WB-MRI alone. Remarkably, 15/18 patients (83%) with lobular tumors had additional metastases discovered, and seven of them had a change in treatment strategy (Figure 1). This can be partly due to the known low metabolic activity of lobular tumors that drastically lowers the accuracy of PET/CT. Additionally, in 13 patients (22%), a diagnostic biopsy was spared due to evidence of progression on WB-MRI.

**Figure 1.**
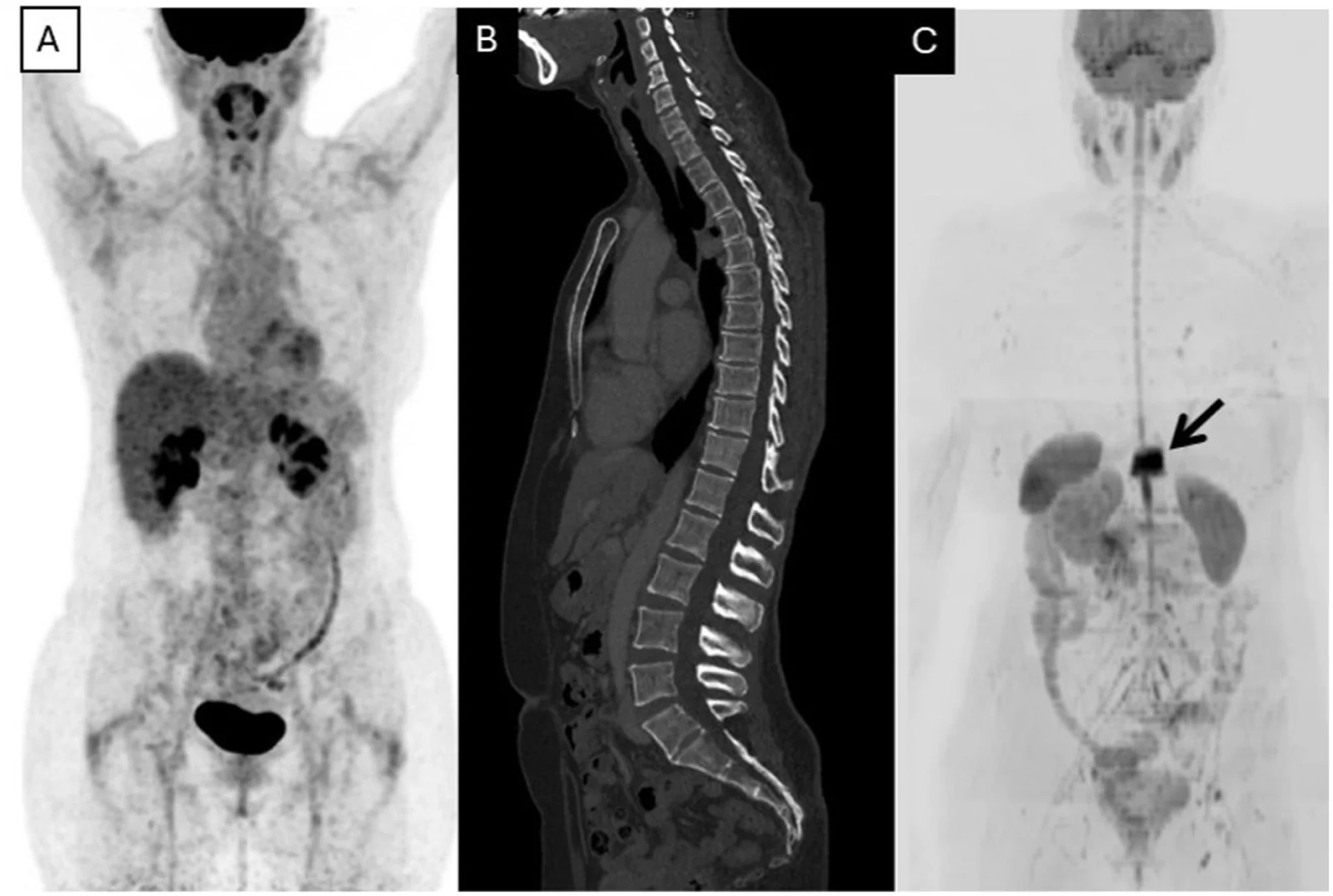
(A) FDG-PET/CT MIP image; (B) Contrast Enhanced (CE) CT sagittal images; (C) Whole-Body MRI MIP image. Figure 1 shows how conventional imaging (FDG-PET/CT and CECT, respectively A and B) demonstrated normal findings in a 53-year-old breast cancer patient with a lobular histotype. However, due to the increase of the tumoral markers patients also underwent a WB-MRI (C) that clearly depicted pathologic bone marrow signal at the vertebral body of T11 (arrow).

Among the 23 patients who underwent at least one reassessment by WB-MRI and SIT, the concordance rate was 69.6% (16 patients; 95% confidence interval 30-64). A total of seven patients (30.4%) showed non-concordant findings; the therapeutic approach was modified in five of these patients (21.7%).

A total of 18 oligometastatic patients at SIT underwent WB-MRI. Of these, nine patients were staged with CT and nine with PET/CT. In 11 cases, only one metastasis was detected by SIT. The majority of patients had HR+/HER2-negative disease (14 patients), three patients were triple-negative, and one patient was HER2-positive. In nine cases (50%), WB-MRI revealed additional metastases (bone metastases in 89% cases) compared to SIT. Finally, in a total of eight patients (44.4%), the OMD was redefined: three patients (16.7%) were reclassified as disease-free, and the other five (27.7%) as polymetastatic (having more than five metastases).

## Discussion

The accurate detection of bone metastases in BC patients is crucial for effective management of the disease. Early detection increases survival, quality of life and reduces treatment costs.^15^ Many models have shown that targeted imaging services offer high benefit-cost ratios. WB-MRI is firmly established as a first-line imaging modality in multiple myeloma, and in cancer predisposition syndromes such as Li-Fraumeni^16^ and there is growing evidence supporting its role in advanced prostate cancer.^17^ In these contexts, WB-MRI has also shown a predictive role.^18^

At present, WB-MRI shows promising results also in BC, but comprehensive sensitivity and specificity data remain limited. Our preliminary results support previously published data. The use of WB MRI may provide better assessment of disease extent and progression in patients with mBC.^19^

Although our study is limited by a small sample size and heterogeneous patient population, WB-MRI emerges as a tool with consistent clinical impact, especially for lobular cancers and OMD, which can be reclassified primarily due to higher sensitivity in detecting additional bone metastases compared to SIT.

WB-MRI should be used to define OMD in clinical practice and should be included in prospective clinical trials investigating OMD management. Furthermore, the potential utility of WB-MRI in staging and follow-up of BC patients, especially in the presence of lobular histology and bone predominant disease, should be further explored.

Prospective studies are warranted to determine whether the use of WB-MRI translates into improved progression-free and overall survival, always considering its potential limitations: it can be more expensive and may not be widely accessible to all patients; the interpretation of WB-MRI results can be complex, and there is a need for standardization in acquisition, interpretation, and reporting findings. Future directions of our study include redefining OMD using circulating biomarkers in addition to WB-MRI, and correlating radiomic data with biomarkers (ctDNA, CTCs, and inflammatory cells) to identify the onset of lesions at an earlier stage.^20^

## Data Availability

The data underlying this article will be shared on reasonable request to the corresponding author.
